# Enhanced Electrochemical Performance of Highly Porous CeO_2_ ‐Doped Zr Nanoparticles for Supercapacitor Applications

**DOI:** 10.1002/jemt.24728

**Published:** 2024-11-07

**Authors:** M. V. Arularasu, T. V. Rajendran, Bassim Arkook, Moussab Harb, K. Kaviyarasu

**Affiliations:** ^1^ Sustainable Energy and Environment Research Unit, Center for Global Health Research, Saveetha Medical College Saveetha Institute of Medical and Technical Science Chennai Tamil Nadu India; ^2^ Department of Chemistry SRM Institute of Science and Technology Chennai Tamil Nadu India; ^3^ Department of Physics, Faculty of Science King Abdulaziz University Jeddah Saudi Arabia; ^4^ UNESCO‐UNISA Africa Chair in Nanoscience's/Nanotechnology Laboratories, College of Graduate Studies University of South Africa (UNISA) Pretoria South Africa

**Keywords:** CeO_2_‐doped Zr, supercapacitor, ultrasonic‐assisted synthesis

## Abstract

The aim of this work was to develop an ultrasonic‐assisted synthesis method for the fabrication of CeO_2_‐doped Zr nanoparticles that would improve the performance of supercapacitor electrodes. This method, which eliminates the need for high‐temperature calcination, involves embedding CeO_2_ into Zr nanoparticles through 1 hr (CeO_2_‐Zr‐1) and 2 hrs (CeO_2_‐Zr‐2) of ultrasonic irradiation, resulting in the formation of nanostructures with significant improvements in their electrochemical properties. Through physicochemical analysis, we observed that the CeO_2_‐doped Zr nanoparticles, particularly those treated for 2 hrs (CeO_2_‐Zr‐2), exhibit superior crystalline phase purity, optimal chemical surface composition, minimal agglomeration with particle sizes below 50 nm, and an impressive average surface area of 178 m^2^/g. Compared to the 1 hr irradiation samples (CeO_2_‐Zr‐1) and undoped CeO_2_ nanoparticles, the (CeO_2_‐Zr‐2) electrodes demonstrated a remarkable capacitance of 198 Fg^−1^ at a current density of 1 A/g while maintaining ~94.9% of their capacity after 3750 cycles. This indicates not only good reversibility but also exceptional stability. In (CeO_2_‐Zr‐2) samples, the nanospherical structure achieved through ultrasonic synthesis is responsible for the enhanced capacitive behavior and stability, along with the synergistic effects caused by Zr doping, which improves the CeO_2_ nanoparticle conductivity to a significant extent. Surface areas of the electrodes are larger due to the combination of these two materials, which contribute to their superior performance.

## Graphical abstract

To achieve an effective strategy for tuning the surface redox behavior of CeO_2_ for the high‐performance supercapacitor, this study fabricates CeO_2_‐doped Zr using an ultrasonic‐assisted method at different temperature processes (1 and 2 hrs). Ultrasonic‐assisted Zr doped with CeO_2_ may display greater crystallinity, pores, and zirconium content than commercial Zr, so that it will perform more efficiently electrochemically. This phenomenon improves the electron transport efficiency, facilitating the charge/discharge behavior and capacitance delivery. In ultrasonic‐assisted CeO_2_‐doped Zr, the specific capacitance was significantly enhanced, and the capacity was well retained at 1 Ag^‐1^ current density. An efficient energy storage application is revealed by the CeO_2_‐Zr‐2 electrode in the brief study.

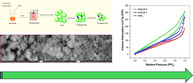

**Summary**

- Ultrasonic‐assisted synthesis method eliminates the need for high‐temperature calcination process.
- Incorporation of Zr into CeO_2_ nanoparticles through 1 hr (CeO_2_‐Zr‐1) and 2 hrs (CeO_2_‐Zr‐2) of ultrasonic irradiation, resulting in the formation of nanostructures with significant improvements in their electrochemical properties.
- The CeO_2_‐Zr‐2 nanoparticles exhibited enhanced dispersion and porosity compared to the other samples, which are conducive to superior conductivity.
- The CeO_2_‐Zr‐2 devices had specific capacitance of 198 Fg^−1^ at current densities of 1 Ag^−1^.

## 1 Introduction

Research related to low‐cost energy production and lightweight and safe storage devices is becoming increasingly essential in modern society due to the rapid consumption of non‐renewable energy resources (Nandi et al. 2020). Recently, energy sources such as wind and solar have become popular worldwide because they are pollution‐free and renewable. Supercapacitors attract tremendous attention owing to high power density, safety, stability, fast charge/discharge speed, and environmental friendliness (Lin et al. 2021; Chen, Zheng, and Zhu 2018). Meanwhile, power‐dense (∼10 kW kg^−1^) supercapacitors with appreciable long cyclic stability (> 1,00,000 cycles) have attracted much attention from the demand for miniaturized electronics. Compared with Li‐ion batteries, supercapacitors have a high energy density, a long‐life cycle, and faster charging ability. However, supercapacitors have lower power density than Li‐ion batteries (Lei et al. 2020; Zhao et al. 2019, 2017). To achieve high performance, supercapacitors require the key challenge of design with a larger surface area, superior mechanical robustness, and good conductivity with sufficient ionic diffusion (Zhou et al. 2019). This is the main reason for recent research on the development of supercapacitor technology. There are two main ways that supercapacitors are created: electrochemical double‐layer capacitors (EDLC) and pseudocapacitors (PC's) (Lee et al. 2018). In energy storage devices, the charge storage process involves the adsorption of ions to the active sites, which is the reason for the transport of charged particles and electrons; therefore, redox reactions occur on the surface of the electrode. Electrochemical double‐layer capacitors store energy using an electrostatic adsorption/desorption mechanism, while Faradaic redox reactions are involved in the pseudocapacitor. Higher storage is achieved by the redox reaction in the pseudocapacitor rate compared to the charge/discharge rates. (Sekhar et al. 2016; Prasanna et al. 2018; Mofarah et al. 2019).

Among pseudocapacitor materials, transition metal oxides, carbon materials, and conductive polymers are promising materials for supercapacitor electrodes (Shokry et al. 2022). So far, active materials such as CuO, V_2_O_5_, CeO_2_, MnO_2_, NiO, and Co_3_O_4_ have been extensively investigated for supercapacitors due to their high specific capacitance and high powder density (Prasanna et al. 2018; Samuel et al. 2019). Mainly due to its easy‐to‐prepare, cost‐effective, environmentally friendly, outstanding redox capacitance performance and corrosion resistance, CeO_2_ is prone to be a promising electroactive redox material (Rabani et al. 2021; Liang et al. 2020; Wang et al. 2019). Furthermore, dual oxidation states (Ce(III) and Ce(IV)) of CeO_2_ offer a new and challenging electrode material for supercapacitor applications. Benefiting from the outstanding redox characteristics of CeO_2_ can enhance the oxygen vacancies and improve materials' surface properties (Nabi et al. 2022; Tan et al. 2014). Increasing the oxygen vacancies and delocalization of electrons can also improve the optical properties, conductivity, and electrochemical properties (Kumar et al. 2018a). However, CeO_2_ has intrinsically low electrical conductivity, which frequently results in low electrochemical activity. Poor conductivity also makes it difficult for the material to engage in the electrochemical storage process, which negatively impacts supercapacitor performance. Various efforts have been made to address this issue, including incorporating transition metal doping and forming composites using metal oxides and conducting polymers (Sharan and Dutta 2017; Sun et al. 2020). Among them, Sr., Ca, Mg, Fe, and Co transition metal‐doped CeO_2_ have been studied, and an efficient strategy to improve significantly the energy density and electron–electron interaction by introducing a surface‐dominated Faradic reaction mechanism (Nabi et al. 2022; Sharan and Dutta 2017).

Numerous approaches are reported for the preparation of CeO_2_, such as hydrothermal, sol–gel, microwave‐assisted, co‐precipitation, microemulsion methods, etc. (Rani, Ahlawat, and Goswami 2020; Tao et al. 2010). The two main physical processes linked to sonochemistry in acoustic cavitation in the liquid phase are nebulization and the development of bubbles that eventually implosively collapse. This pathway facilitates enhanced solute diffusion, which in turn causes the product to exhibit a narrow size distribution, a rapid rate of reaction, and better porosity without requiring a high‐temperature crystallization procedure. Moreover, it is readily scalable to meet industrial requirements (Huang et al. 2011). Moreover, surface porosity has many advantages for the performance of electrochemical devices and supercapacitors. More porosity can transfer the doped ions, significantly reducing the ion‐transport resistance, improving the charge–discharge rate, and enhancing the cyclic stability of electrochemical devices.

On the basis of the above considerations, in this work, CeO_2_‐doped Zr is fabricated by the ultrasonic‐assisted method at different time processes (1 hr & 2 hrs) to achieve an effective strategy to tune the surface redox behavior of CeO_2_ for the high‐performance supercapacitor. As‐ultrasonic‐assisted obtained, CeO_2_‐doped Zr possesses a high crystallinity, high pore‐size distribution, and high zirconium content, which may result in superior electrochemical performance. Astonishingly, the inductive effect increases the electron transport efficiency, facilitating the capacitance delivery and charge/discharge behavior. Ultrasonic‐assisted CeO_2_‐doped Zr exhibited a significantly enhanced specific capacitance of 198 Fg^−1^ at a current density of 1 Ag^−1^, excellent cyclic stability, and capacity retention. The brief study proves that the CeO_2_‐Zr‐2 electrode reveals that it has an efficient energy storage application.

## 2 Experimental Method

### 2.1 Synthesis CeO_2_ ‐Doped Zr Nanoparticles

**SCHEME 1:**
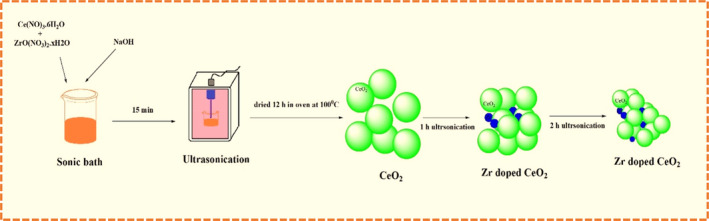
Mechanism of preparation of CeO_2_ doped Zr nanoparticles.

The synthesis of pure CeO_2_ nanoparticles and CeO_2_‐doped Zr nanoparticles was obtained using a single‐step ultrasonic‐assisted synthesis method, and the CeO_2_‐doped Zr nanoparticle's size and morphology were tuned as a function of ultrasonic irradiation time. Cerium nitrate hexahydrate [Ce(NO)_3_·6H_2_O], zirconium nitrate [ZrO(NO_3_)_2_·*x*H_2_O], and sodium hydroxide (NaOH) were used. During the production of CeO_2_‐doped Zr nanoparticles, 4.3 g and 2.3 g of [Ce(NO)_3_·6H_2_O] and [ZrO(NO_3_)_2_·*x*H_2_O] were dispersed in 50 mL of ethanol and placed in a sonic bath for 15 min. A dropwise addition of 4.0 g of NaOH was made to the solution above during the sonication process. In addition, a yellow suspension was produced by adding 20 mL of DI water to the previously mentioned solution. The precursor solution was then subjected to an ultrasonic irradiation technique for one and a half hours at a frequency of 20 kHz and 500 W (Sonics‐VCX 500, Taiwan, per cycle 20' s). After washing in deionized and distilled water, the final CeO_2_‐doped Zr nanoparticles were obtained. They were then vacuum dried for 12 hrs at 100°C. The synthesis of pure CeO_2_ nanoparticles followed the same procedure without zirconium nitrate with 1 h ultrasonic irradiation (*the preparation process of CeO*_*2*_ *doped Zr nanoparticles is outlined in* Scheme 1).

### 2.2 Instrumentation

An FESEM (HITACHI S‐3000 N, Japan) was used in conjunction with an energy‐dispersive spectrometer (EDX) was used to determine the morphological and elemental composition of the materials at an accelerating voltage of 0.3–30 kV. The crystalline phase, crystalline size, and formation of synthesized materials are ascertained by XRD (Shimadzu XRD 6000, Japan) examination utilizing CuK_α_ radiation 1.5406 Å. The functional group analysis of the samples was measured with PerkinElmer fourier transforms infrared spectroscopy (JASCO FT/IR‐6600 spectrometer). The nitrogen adsorption–desorption measurement was obtained using BET method (GEMINI VII 2390).

### 2.3 Preparation of Electrode for Electrochemical Measurements

(CeO_2_‐Zr‐1) and (CeO_2_‐Zr‐2) nanoparticles electrochemical supercapacitor properties were investigated at room temperature (RT) using a Biologic VSP‐300 Potentiostat device. In a conventional three‐electrode system, Ag/AgCl and Pt electrodes were used as the reference and counter electrodes. A binder was weighed and mashed using a pestle to create the working electrode, which was composed of ~75% manufactured compound (*active material*), ~15% carbon black, and ~10% polyvinylidene fluoride (*PVDF*). Furthermore, *N*‐methyl pyrrolidone (NMP) drops were added to obtain the uniform slurry. In order to prepare the graphite sheet current collector for future work, the resulting slurry was thus applied to both sides of its surface with dimensions of 1 × 0.5 cm, 0.3 m length, and thickness, respectively. It was then dried for 12 hrs at about 80°C. In 1 M KOH electrolyte solution, cyclic voltammetry (CV) analysis was carried out at potentials ranging from −0.2 V to 0.8 V using scan rates of 10, 20, 30, 50, 70, and 100 mVs^−1^; galvanostatic charge and discharge (GCD) was carried out at 1 Ag^−1^.

## 3 Results and Discussion

### 3.1 Structural Properties

**FIGURE 1:**
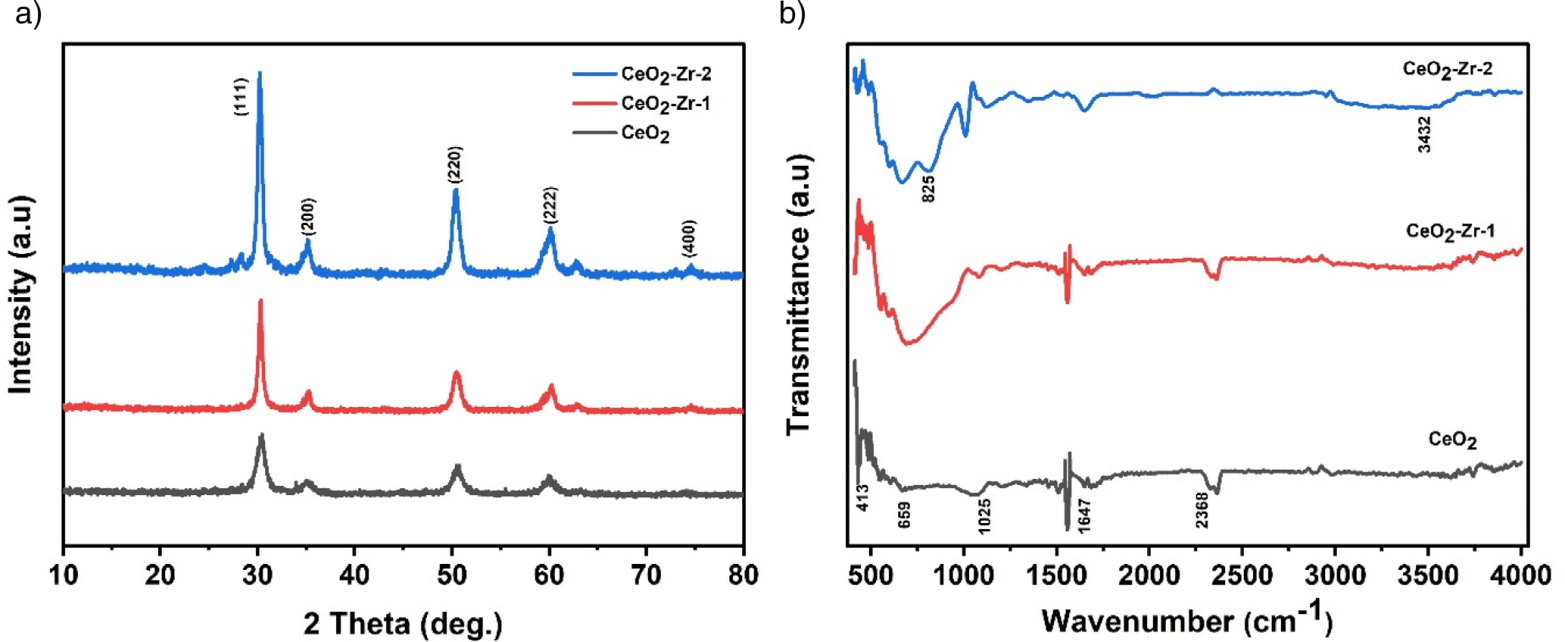
(a) XRD pattern; (b) FTIR spectrum of CeO_2_ nanoparticles, CeO_2_‐Zr‐1, CeO_2_‐Zr‐2.

The structural properties of pure CeO_2_ nanoparticles and their Zr‐doped counterparts were comprehensively evaluated using X‐ray diffraction (XRD) analysis. This investigation aimed to elucidate the effects of Zr doping and ultrasonic irradiation on the crystalline structure of CeO_2_ nanoparticles. The analysis revealed distinct diffraction peaks at 2θ = 29.8^o^, 34.3^o^, 49.1^o^, 60.2^o^ and 74.4^o^ corresponding to the (111), (200), (220), (222), and (400) planes of the face‐centered cubic (*fcc*) structure of CeO_2_ (JCPDS: 34–0394), across all samples, including CeO_2_, CeO_2_‐Zr‐1 (*1 h of Zr doping* via *ultrasonic irradiation*), and CeO_2_‐Zr‐2 (*2 h of Zr doping* via *ultrasonic irradiation*), as depicted in Figure 1a. Remarkably, the XRD patterns did not exhibit any separate peaks for ZrO_2_, suggesting the successful incorporation of Zr^4+^ ions into the CeO_2_ lattice without forming separate ZrO_2_ phases. This incorporation was further evidenced by the enhanced intensity of the diffraction peaks for the CeO_2_‐Zr‐2 sample, indicating an increase in crystallinity attributed to the Zr doping and extended ultrasonic irradiation time. Quantitative analysis using the Scherrer equation revealed a significant reduction in the average crystallite size from 27.3 nm in pure CeO_2_ nanoparticles to 18.5 nm and 11.7 nm in (CeO_2_‐Zr‐1) and (CeO_2_‐Zr‐2), respectively. Zr doping and ultrasonic irradiation are credited for this reduction, suggesting that these modifications enhance crystallinity and reduce crystallite size simultaneously (Chen et al. 2010; Kaviyarasu et al. 2016, 2017). The observed structural modifications have profound implications for the electrochemical performance of these materials in supercapacitor applications. Specifically, the increase in crystallinity and reduction in crystallite size is anticipated to enhance the electrochemical properties of CeO_2_ based supercapacitors, including their capacitance, rate capability, and cycling stability. These enhancements are attributed to the improved conductivity and increased surface area provided by the nano‐spherical morphology and the optimized Zr doping facilitated by the ultrasonic synthesis method.

### 3.2 FTIR Analysis

FTIR spectroscopy was utilized to elucidate the interface interactions and identify the functional groups on CeO_2_ nanoparticles and their Zr‐doped variants (CeO_2_‐Zr‐1 and CeO_2_‐Zr‐2). Figure 1b illustrates the FTIR spectra for these samples, showcasing distinct peaks that reveal the chemical nature of these materials. The peak observed at ~3432 cm^−1^ is associated with the O–H stretching vibrations, indicative of water molecules on the surface of the (CeO_2_‐Zr‐2) nanoparticles. This observation suggests the potential for water molecule interactions with the nanoparticle surfaces, which could play a role in the electrochemical behavior of these materials in supercapacitor applications. The C=O stretching vibration at 1647 cm^−1^, the Ce–O–Ce stretching vibration at 659 cm^−1^, the O–O band at 1025 cm^−1^, the CH_2_ vibration at 2368 cm^−1^, and the Ce–O stretching vibration at 413 cm^−1^ and some of the other peaks that have been identified. These assignments include information about the molecular structure of the samples and are in line with values found in the literature. Zr has been successfully doped into the CeO_2_ lattice as evidenced by the more intense peak at 659 cm^−1^ in the doped samples and the emergence of a new peak at 825 cm^−1^ in the CeO_2_‐Zr‐2 sample, which is attributable to Zr‐O stretching vibrations.

These findings are corroborated by references to the literature where similar vibrational modes have been reported, affirming the accuracy of these assignments. Furthermore, the observed functional groups, particularly those indicating organic remnants like CH_2_ and C=O, prompt a discussion on their origin—whether from the precursors used or as part of surface modifications—and their potential impact on the electrochemical performance of the nanoparticles. The presence of surface‐adsorbed water, as evidenced by the O–H stretching vibrations, raises questions about the role of these molecules in ionic transport or capacitance.

### 3.3 Morphology and BET Analysis

**FIGURE 2:**
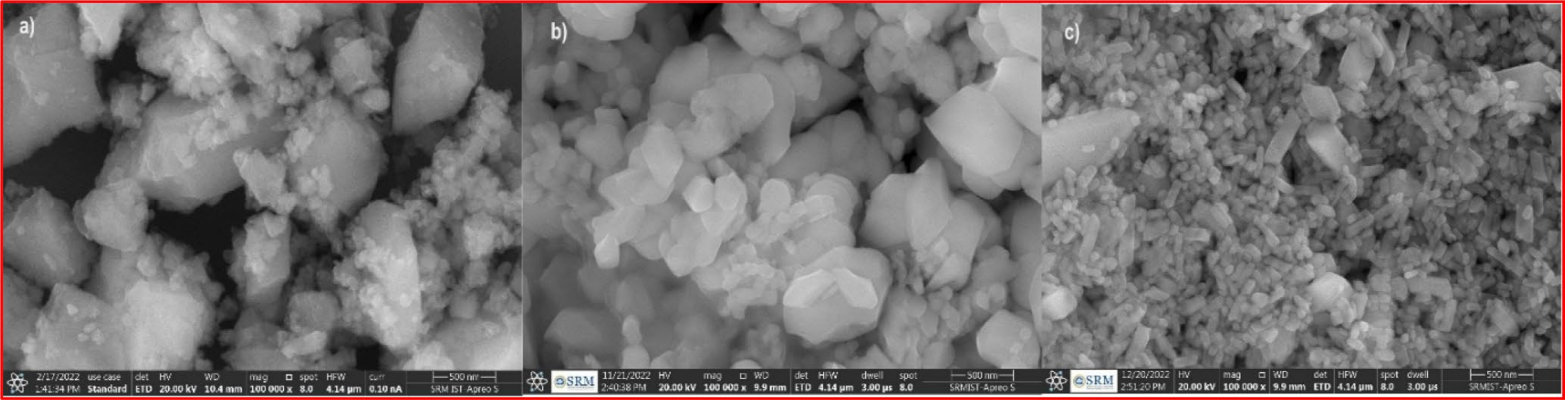
FESEM images of (a) CeO_2_ nanoparticles; (b) CeO_2_‐Zr‐1; (c) CeO_2_‐Zr‐2 composite.

The morphological characteristics of CeO_2_ nanoparticles and their Zr‐doped variants (CeO_2_‐Zr‐1 and CeO_2_‐Zr‐2) were thoroughly analyzed using field emission scanning electron microscopy (FESEM) and Transmission Electron Microscopy (TEM), complemented by selected area electron diffraction (SAED), and energy‐dispersive X‐ray spectroscopy (EDAX) for a comprehensive understanding of the structural changes and elemental composition. FESEM images as shown in Figure 2 revealed a distinct transformation in the surface morphology with Zr doping. As a result of their high surface area, pristine CeO_2_ nanoparticles showed a jagged surface with clusters of small spheres. When (CeO_2_‐Zr‐1) and (CeO_2_‐Zr‐2) are synthesized with ultrasonic‐assisted synthesis, a more uniform sphere‐like shape with particle diameters under 50 nm is achieved (Jayakumar, Albert Irudayaraj, and Dhayal Raj 2022; Mobeen Amanulla, Maria Magdalane, and Saranya 2021; Maria Magdalane, Siddhardha, and Ramalingam 2021). This uniformity is expected to facilitate ion transport, potentially enhancing the electrochemical performance. The presence of larger particles amidst smaller ones in the (CeO_2_‐Zr‐2) sample directly indicates Zr incorporation, suggesting successful doping. A quantitative analysis, including particle size distribution and porosity assessment, would enrich understanding how these morphological changes contribute to the material's electrochemical properties.

**FIGURE 3:**
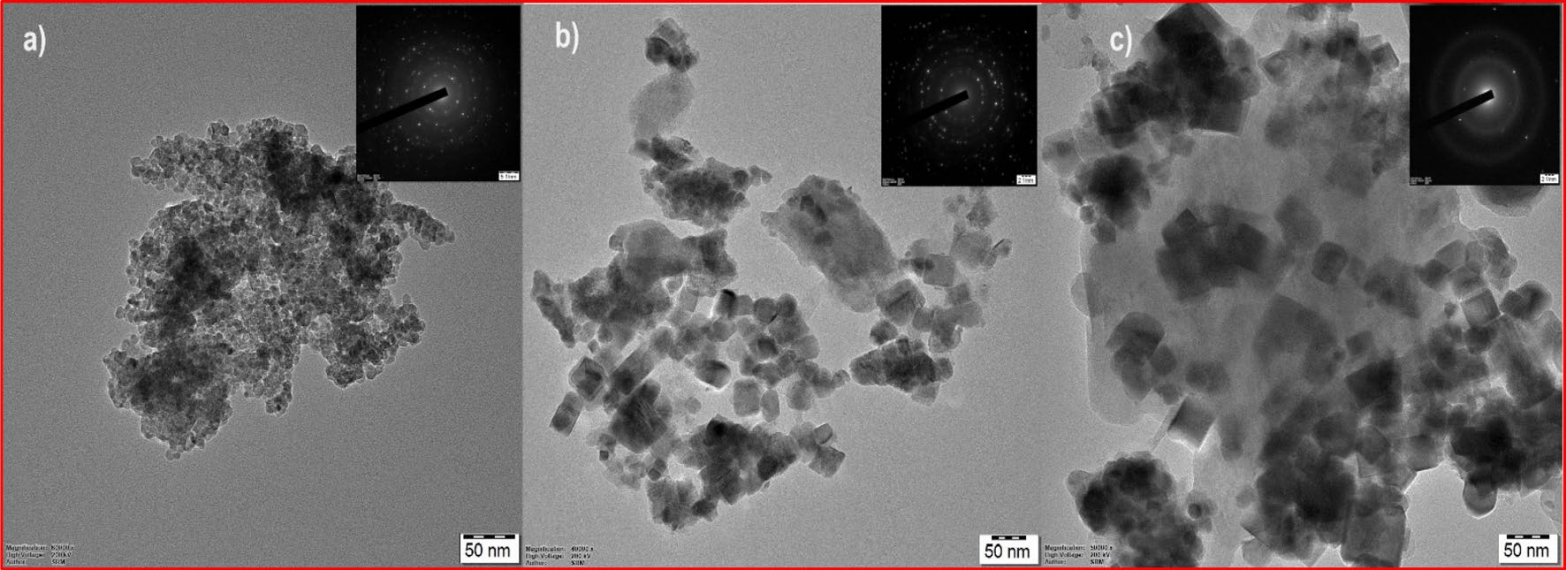
TEM images of (a) CeO_2_ nanoparticles; (b) CeO_2_‐Zr‐1; (c) CeO_2_‐Zr‐2 composite.

**FIGURE 4:**
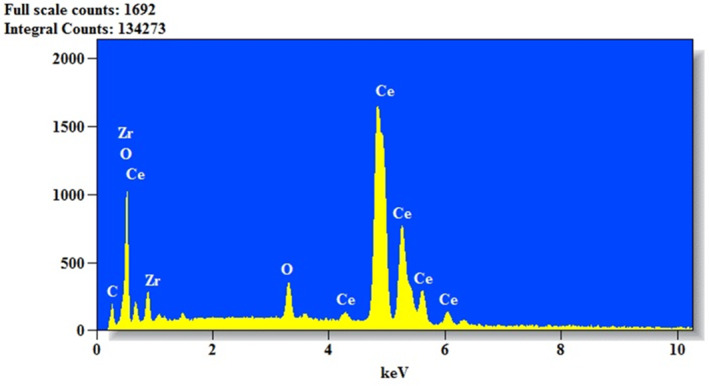
EDX spectrum of CeO_2_‐Zr‐2 nanocomposite.

**FIGURE 5:**
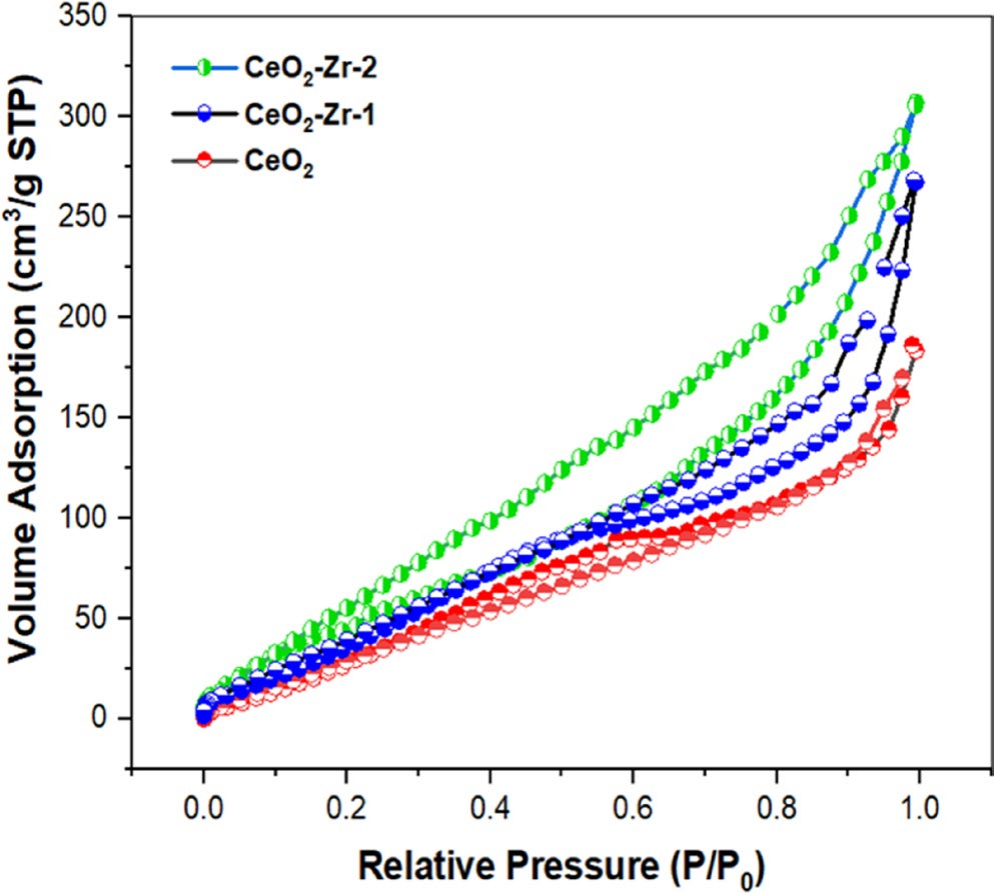
BET spectrum of CeO_2_ nanoparticles, CeO_2_‐Zr‐1, CeO_2_‐Zr‐2 composite.

TEM images as shown in Figure 3a,b imaging provided further insights into the nanostructure, revealing a network‐like arrangement in the pristine CeO_2_, with a significant reduction in agglomeration and an increase in porosity in the Zr‐doped samples. The (CeO_2_‐Zr‐2) nanoparticles exhibited enhanced dispersion and porosity compared to the other samples, which are conducive to superior conductivity. The SAED patterns, as shown in the inset of Figure 3, corroborated the crystalline nature of the nanoparticles, displaying randomly scattered diffraction spots and ring patterns. A detailed analysis correlating these diffraction patterns with the crystallinity and phase purity, especially in Zr doping, would be beneficial. EDAX analysis confirmed the elemental composition of the (CeO_2_‐Zr‐2) sample, detecting peaks corresponding to Ce, Zr, O, and C without any impurities, which validates the doping process as shown in Figure 4. Discussing the stoichiometry and potential effects of detected elements on the nanoparticles' electrochemical behavior could provide deeper insights. Figure 5 represent the nitrogen adsorption–desorption isotherms revealed a mesoporous structure for all samples, with the BET surface area increasing from 147 m^2^/g for pristine CeO_2_ to 178 m^2^/g for (CeO_2_‐Zr‐2). Increasing the surface area of a metal by doping it with Zr and ultrasonically treating it results in a greater specific capacitance and improved electrochemical performance. By examining the pore size distribution and volume, and how they influence ion transport and storage, we can strengthen this relationship between morphological characteristics and electrochemical activity (Sabarinathan et al. 2023; Srinivasan, Uthiram, and Punithavelan 2023; Rasupillai Dharmaraj et al. 2023; Jayakumar and Albert Irudayaraj 2024; Mobeen Amanulla and Sundaram 2019).

### 3.4 Electrochemical Studies

#### 3.4.1 Cyclic Voltammetry

**FIGURE 6:**
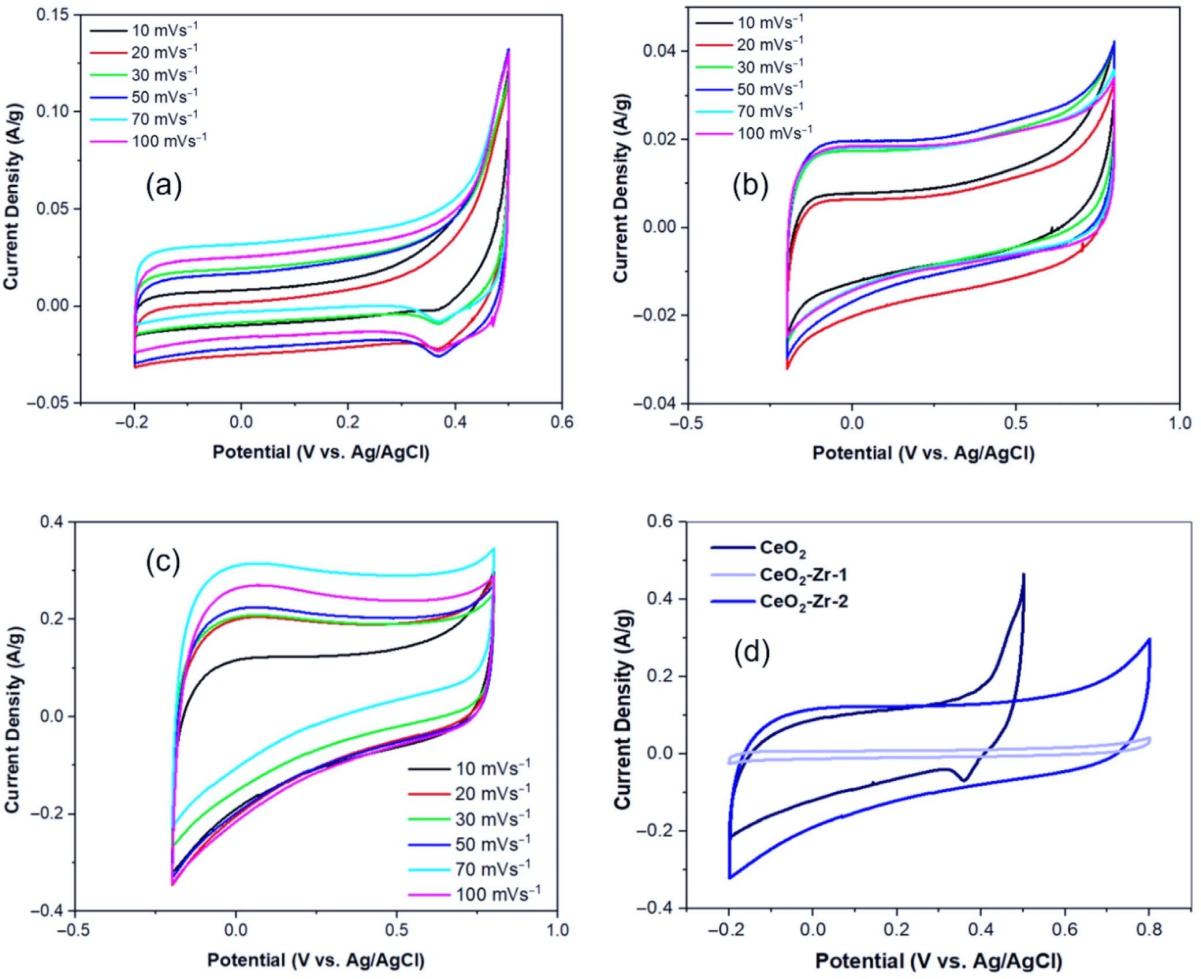
CV curves of (a) CeO_2_ nanoparticles; (b) CeO_2_‐Zr‐1; (c) CeO_2_‐Zr‐2 at different scan rates between 5–100 mVs^−1^; and (d) comparison of CV curves of CeO_2_ nanoparticles, CeO_2_‐Zr‐1, CeO_2_‐Zr‐2 at a scan rate of 10 mVs^−1^.

The CV study of CeO_2_ nanoparticles and their Zr‐doped variants, (CeO_2_‐Zr‐1 and CeO_2_‐Zr‐2), was performed across a potential window of −0.5 to 0.8 V at varying scan rates from 5 to 100 mVs^−1^, as shown in Figure 6a–c. This analysis yielded significant insights into the electrochemical behavior of these materials, revealing redox peaks that underscore their pseudocapacitive capabilities, primarily attributed to the valence state transitions of cerium (Ce^3+^/Ce^4+^). Notably, the CV curves retained their shape across the range of scan rates for all samples, indicating their excellent structural integrity and capacitive performance. Further examination revealed that (CeO_2_‐Zr‐2) exhibited the largest area under the CV curves at a scan rate of 10 mVs^−1^, as illustrated in Figure 6d, suggesting superior capacitance compared to (CeO_2_‐Zr‐1) and the pristine CeO_2_ nanoparticles. This observation led to a focus on the doped samples for subsequent investigations. The increased specific capacitance in the Zr‐doped samples is attributed to enhancements in surface area and electrical conductivity, which are crucial for improved ion transport and electrochemical reactivity.

Quantitative analysis of the specific capacitance values derived from the CV curves would facilitate direct comparison between the samples, providing a clearer understanding of the impact of Zr doping and ultrasonic irradiation. This analysis is essential for evaluating the materials' suitability for supercapacitor applications, particularly in contexts requiring rapid charge/discharge cycles. The study discusses the Faradaic redox processes facilitated by Zr incorporation to offer a comprehensive perspective on the electrochemical mechanisms (Ponkumar, Prakashbabu, and Parasuraman 2024; Dillip et al. 2016). These processes are integral to the observed pseudocapacitive behavior, with Zr doping potentially altering the electronic structure of CeO_2_, thereby enhancing its conductivity and ion transport capabilities. Addressing the stability and durability of the electrodes, the study plans to conduct extended cycling tests. These tests are pivotal for assessing the long‐term performance of the CeO_2_‐doped Zr electrodes and ensuring their practical applicability in energy storage technologies.

$${\mathrm{Ce}}^{\mathrm{IV}}O_{2}+K^{+}+e^{-}⇌{\mathrm{Ce}}^{\mathrm{III}}O.\mathrm{OK}$$

#### 3.4.2 Galvanostatic Charge/Discharge and EIS Studies

**FIGURE 7:**
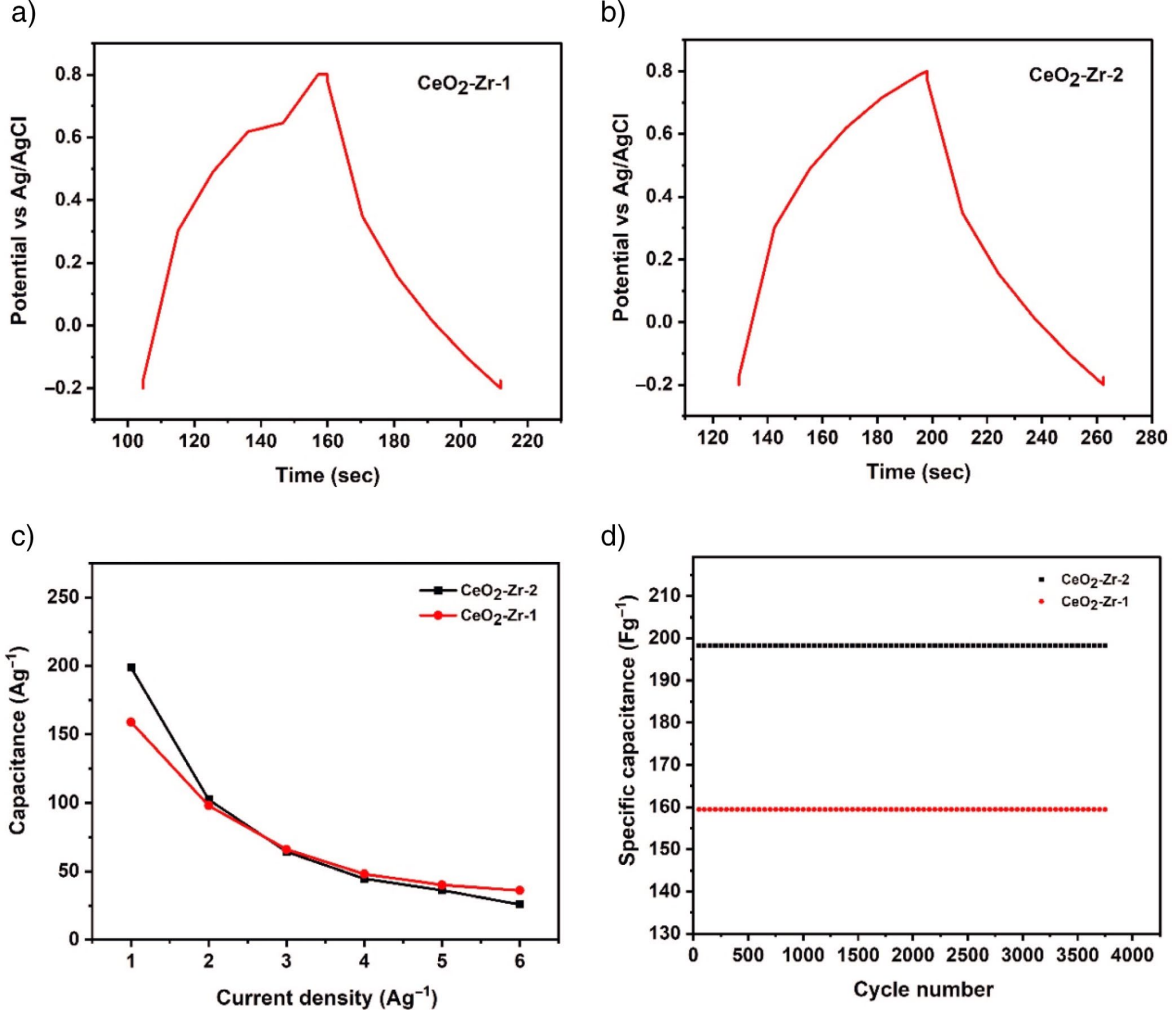
GCD curves of (a) CeO_2_‐Zr‐1; (b) CeO_2_‐Zr‐2; (c) Specific capacitance of CeO_2_‐Zr‐1 CeO_2_‐Zr‐2 at different current densities and (d) cyclic performance of CeO_2_‐Zr‐1 and CeO_2_‐Zr‐2 at a current density of 1Ag^−1^.

To know the charge‐storage efficiency and long‐term cyclic stability of the (CeO_2_‐Zr‐1 and CeO_2_‐Zr‐2) supercapacitor, GCD analysis was performed within −0.2 to 0.8 V at a current density of 1Ag^−1^ and the results as shown in Figure 7a,b. As per GCD studies, all the electrodes exhibit an asymmetric shape along with the non‐quasi triangle shape, representing a pseudocapacitive behavior consistent with the CV curves. The GCD comparison curve of the (CeO_2_‐Zr‐1 and CeO_2_‐Zr‐2) electrodes; as expected, the GCD curve of a CeO_2_‐Zr‐2 electrode shows the expanded area of the GCD curve when compared with CeO_2_‐Zr‐1 electrode (Chuni et al. 2018; Dhivya Angelin et al. 2020). The following formula was used to determine the specific capacitance: C_sp_ is equal to I × ∆t/m X ∆v(I), where m is the active material's mass (g), I is the current (A), ∆t is the discharging time (s), and ∆V is the potential (V) (Sun et al. 2020). The calculated specific capacitance based on the GCD values of CeO_2_‐Zr‐1 and CeO_2_‐Zr‐2 samples to be 159 Fg^−1^ and 198 Fg^−1^, respectively, at the current density of 1 Ag^−1^ as shown in Figure 7c. Noteworthily, the CeO_2_‐Zr‐2 electrode possessed the highest specific capacitance 198 Fg^−1^ at 1Ag^−1^, which can be attributed to the following factors: (i) Zr‐doping can effectivity utilize their electrochemical activity (Elanthamilan et al. 2017; Sakthivel et al. 2018; Chen et al. 2017), (ii) ultrasonic irradiation time of the CeO_2_‐Zr‐2 boost the conductivity (iii) during the charge/discharge process the electron charge transfer occurs that concomitant to the insertion/extraction of Zr ions into for cerium oxide lattice, and (iv) high surface area of CeO_2_‐Zr‐2 enables fast electron transfer in the electrode. The CeO_2_‐Zr‐2 devices had specific capacitance of 198 Fg^−1^ at current densities of 1Ag^−1^. At all current densities, the specific capacity of CeO_2_‐Zr‐2 decreases slowly, indicating its good rate performance. These results demonstrate that the current synthesis technique can provide a high‐performance supercapacitor and be used in energy storage and conversion applications. Figure 7d depicts the long‐term cycle property of the (CeO_2_‐Zr‐1 and CeO_2_‐Zr‐2) samples at 2 Ag^−1^ within the suitable potential range of −0.2 to 0.8 V. These results show that CeO_2_‐Zr‐2 is a slight decrease in the retention value and, after 3750 charge–discharge cycles, around ~94.9% of initial capacitance. Likewise, the specific capacitance of CeO_2_‐Zr‐1 was at 159 Fg^−1^ after 3750 charge–discharge cycles, resulting in a ~93.5% capacitance retention (Xiaoqi et al. 2020; Rajkamal et al. 2025). The comparison of specific capacitance of previously reported materials and CeO_2_‐Zr‐1 is presented.

**FIGURE 8:**
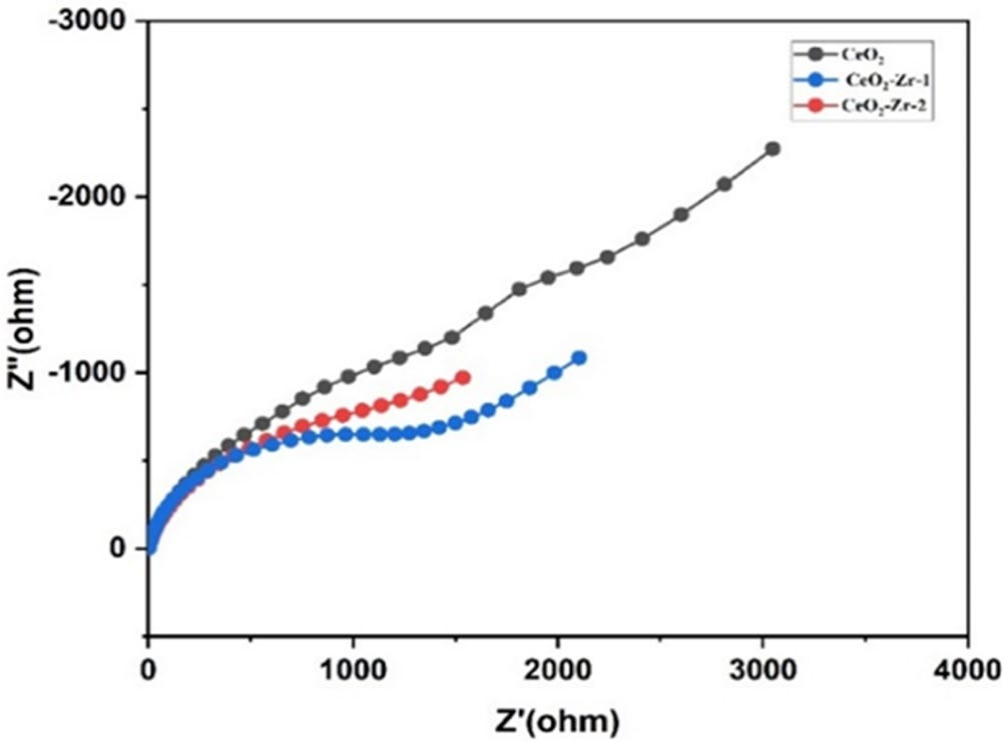
EIS of CeO_2_, CeO_2_‐Zr‐1, and CeO_2_‐Zr‐2 nanoparticles.

To analyze the samples’ capacitive behavior and ion‐transport performance, electrochemical impedance spectroscopy (EIS) was performed (Venkatachalapathy et al. 2024; Kaviyarasu and Madhavan 2024). Figure 8 illustrates the Nyquist plot from EIS of CeO_2_ nanoparticles, CeO_2_‐Zr‐1, and CeO_2_‐Zr‐2 electrode materials. As observed in Figure 8, the Nyquist plot of bare CeO_2_ nanoparticles showed a semicircle with a large diameter compared to the other modified electrode, representing a large charge transfer resistance (*R*_*ct*_). However, the *R*_*ct*_ values of (CeO_2_‐Zr‐1 and CeO_2_‐Zr‐2) significantly decrease respectively, suggesting the redox reaction occurs successfully at the electrode surface. Obviously, CeO_2_‐Zr‐2 possesses lower *R*_*ct*_ at the electrode–electrode interface, indicating that this electrode has better charge transfer ability due to pores in the material, which could shorten the shuttle distance of the ions (Kumar et al. 2018b; Heydari and Gholivand 2017).

## 4 Conclusion

Through the utilization of an ultrasonic‐assisted synthesis method, we have innovatively developed CeO_2_ doped Zr nanoparticles characterized by a highly porous structure tailored for advanced applications of supercapacitors. This research underscores the significant electrochemical enhancements achieved with the optimized CeO_2_‐Zr‐2 sample, which exhibits remarkable specific capacitance (198 Fg^−1^ at a current density of 1 Ag^−1^) in a KOH electrolyte alongside superior long‐term stability, outperforming its precursor, CeO_2_‐Zr‐1. The observed increase in conductivity and reduction in impedance for the CeO_2_‐Zr‐2 variant is attributed to the enhanced ion diffusion facilitated by the material's mesoporous structure, which acts as an effective ion reservoir during the charge–discharge (GCD) cycles. These findings demonstrate the pivotal role of Zr doping in improving the electrochemical properties of CeO_2_ nanoparticles and highlight the potential of such nanostructures in boosting the performance of energy storage devices. The CeO_2_‐doped Zr nanoparticles, particularly the CeO_2_‐Zr‐2 formulation, emerge as promising materials for energy storage and conversion applications thanks to their excellent capacitance, enhanced ion transport efficiency, and robust electrochemical stability. This study paves the way for further exploration and optimization of metal oxide‐based nanostructures for high‐performance supercapacitors, contributing to the advancement of sustainable energy technologies.

## Author Contributions

**M. V. Arularasu:** conceptualization, investigation, visualization, validation, methodology, formal analysis, project administration, writing – review and editing, supervision, resources, software, writing – original draft, data curation, funding acquisition. **T. V. Rajendran:** methodology, validation, visualization, conceptualization, formal analysis, project administration, supervision, data curation, writing – review and editing, investigation. **Bassim Arkook:** visualization, validation, formal analysis, resources, data curation, writing – review and editing, software, investigation, conceptualization, methodology. **Moussab Harb:** funding acquisition, conceptualization, methodology, validation, visualization, data curation, resources, formal analysis, software. **K. Kaviyarasu:** validation, visualization, investigation, formal analysis, software, funding acquisition, methodology, writing – review and editing, project administration, data curation, supervision, resources.

## Ethics Statement

A component of the research process, whose purpose is to protect both the researcher and the participants in the research, which should have their dignity, rights, safety, and welfare respected. In the present study, no animal testing was conducted.

## Conflicts of Interest

This manuscript or a very similar manuscript has not been published, nor is under consideration by any other journal. All authors have seen and approved the final, submitted version of this manuscript.

## Data Availability

The datasets generated during and/or analyzed during the current study are available from the corresponding author on reasonable request. The authors declare no conflicts of interest.
